# DLSTM-Based Successive Cancellation Flipping Decoder for Short Polar Codes

**DOI:** 10.3390/e23070863

**Published:** 2021-07-06

**Authors:** Jianming Cui, Wenxiu Kong, Xiaojun Zhang, Da Chen, Qingtian Zeng

**Affiliations:** 1 School of Computer Science and Engineering, Shandong University of Science and Technology, Qingdao 266510, China; cuijm399@163.com (J.C.); kwx1836092326@163.com (W.K.); chenda@sdust.edu.cn (D.C.); qtzeng@sdust.edu.cn (Q.Z.); 2 State Key Laboratory of High-End Server and Storage Technology, Jinan 250101, China

**Keywords:** polar codes, 5G, SC decoding, bit flipping, LSTM, robustness

## Abstract

Polar code has been adopted as the control channel coding scheme for the fifth generation (5G), and the performance of short polar codes is receiving intensive attention. The successive cancellation flipping (SC flipping) algorithm suffers a significant performance loss in short block lengths. To address this issue, we propose a double long short-term memory (DLSTM) neural network to locate the first error bit. To enhance the prediction accuracy of the DLSTM network, all frozen bits are clipped in the output layer. Then, Gaussian approximation is applied to measure the channel reliability and rank the flipping set to choose the least reliable position for multi-bit flipping. To be robust under different codewords, padding and masking strategies aid the network architecture to be compatible with multiple block lengths. Numerical results indicate that the error-correction performance of the proposed algorithm is competitive with that of the CA-SCL algorithm. It has better performance than the machine learning-based multi-bit flipping SC (ML-MSCF) decoder and the dynamic SC flipping (DSCF) decoder for short polar codes.

## 1. Introduction

Polar codes have been proven to achieve channel capacity [1], thus were selected as the coding standard for the control channels of enhanced mobile broadband (eMBB) scenarios in 2016 [2]. Normally, the block length of the control channel is short or moderate, but Arıkan [3] proved that channel polarization occurs only when the number of channels gradually becomes larger. Therefore, the performance of polar codes is not satisfactory at short or moderate block length due to insufficient channel polarization.

The successive cancellation (SC) decoding [3] has a regular-recursive structure, but the performance is uncompetitive under the finite block length. In order to improve error-correction performance, the succession cancellation list (SCL) and the successive cancellation flipping (SC flipping) algorithms are introduced. Among them, Niu et al. proposed a cyclic redundancy check (CRC) aided SCL algorithm (CA-SCL) [4], which bridges the gap between SC and maximum-likelihood algorithms. The CA-SCL decoding improves performance compared to the conventional SCL decoding [5], but both algorithms come at the cost of high computational complexity. The SC flipping algorithm [6] can achieve a similar computational complexity as the SC decoding, which competes with the CA-SCL decoding. The flipping algorithm constructs the flipping set by the absolute value of log-likelihood ratio (LLR) to locate the first error bit, while there are additional error bits in subsequences. On this basis, a multi-bit flipping algorithm [7,8,9] was proposed to correct multiple error bits. In [8], Zhang et al. defined a set of flipping positions as the critical set (CS). The LLR value is used to determine the flipping bit, which requires sorting the LLR sequence.

Since deep learning was adopted to locate the error bit more accurately than traditional methods [6,7,8,9], many scholars have applied the neural network to decoders [10,11,12]. In [13,14], a decoder based on the convolutional neural network (CNN) is proposed to lower the bit error rate (BER). Chun-Hsiang Chen et al. applied the long short-term memory (LSTM) network to the decoder [15]. The network can efficiently extract features from the decoding and improve the accuracy of error bit prediction, but the complexity increases with the list size. In order to obtain a better flipping strategy, both the LSTM and the reinforcement learning (RL) are applied to the decoder. In [16], the LSTM network predicts the first error bit and the RL identifies other error bits or undoes the previous error flipping. Similarly, a machine learning-based multi-bit flipping SC decoding scheme (ML-MSCF) was proposed by Author17 [17], which uses both the LSTM network and the Q-Learning algorithm to achieve multi-bit flipping. However, it needs a well-trained Q-table to select the flipping bit.

The accuracy of the flipping bit greatly affects the performance of the SC flipping decoder. However, the current SC flipping works do not perform well with short codewords. The LSTM networks have achieved excellent performance in many fields [18,19]. Thus, we propose a double long short-term memory (DLSTM) network to predict the error bits. The main contributions are as follows:

- A DLSTM-based SC flipping decoder is proposed. In addition, all frozen bits are clipping in the output layer to enhance accuracy. We construct the flipping set of the first error bit according to the probability of the network output in descending order, and attempt one-bit flipping.
- By exploring the reliability of bit channel, we propose a novel multi-bit flipping scheme for sequences that reach the maximum number of one-bit flipping and still fail the CRC detector. The candidate bits are sorted in ascending order in terms of the reliability, and the unreliable bits are selected in priority to multi-bit flipping.
- In order to make the proposed algorithm robust, we design the DLSTM network architecture to be compatible with multiple block lengths. We adopt a padding strategy to maintain data integrity, so that the training is not limited by the block length. A masking method is taken to skip the timestep and eliminate the effect of padded invalid data. Simulation results show that the proposed decoding scheme has better error-correction performance than the ML-MSCF decoding and DSCF decoding for short block lengths. It can approach the performance of CA-SCL (*L* = 8).

The remainder of the paper is organized as follows. Section 2 introduces polar codes, SC flipping decoder, and LSTM network. The proposed decoding scheme is presented in Section 3. Section 4 analyzes the decoding performance. Conclusions are drawn in Section 5.

## 2. Preliminary

In this paper, we use the calligraphic characters, such as X, to denote sets. We write *x*, x, and X to denote a scalar, a vector, and a matrix, respectively. In this section, we first review the basic of polar codes. Then, we briefly introduce the SC flipping decoder and LSTM network.

### 2.1. Polar Codes

A polar code of length N with K information bits can be represented by P(N, K). The code rate is R=K/N, 0<K<N. The *K* most reliable channels are selected to transmit the information bits and the remaining N−K channels transmit the frozen bits. The set of information bit indices and frozen bit indices are denoted by A and its complement $A^{C}$. The sequence $u_{1}^{N}=\left\{u_{1},u_{2},\cdots ,u_{N}\right\}$ is obtained by mixing the information bits $u_{A}$ and the frozen bits $u_{A^{C}}$, and a polar codeword $x_{1}^{N}$ is generated by generator matrix $G_{N}$:

(1) $$\begin{array}{c} x_{1}^{N}=u_{1}^{N}G_{N}, \end{array}$$

where $x_{1}^{N}=\left\{x_{1},x_{2},\cdots ,x_{N}\right\}$, $G_{N}=B_{N}F^{⊗n}$, $n=\log_{2}N$, $B_{N}$ is a N×N bit-reversal permutation matrix, and $F^{⊗n}$ denotes the *n*th Kronecker power of F, with

(2) $$F=\left[\begin{array}{cc} 1 & 0\\ 1 & 1 \end{array}\right].$$

**Figure 1:**
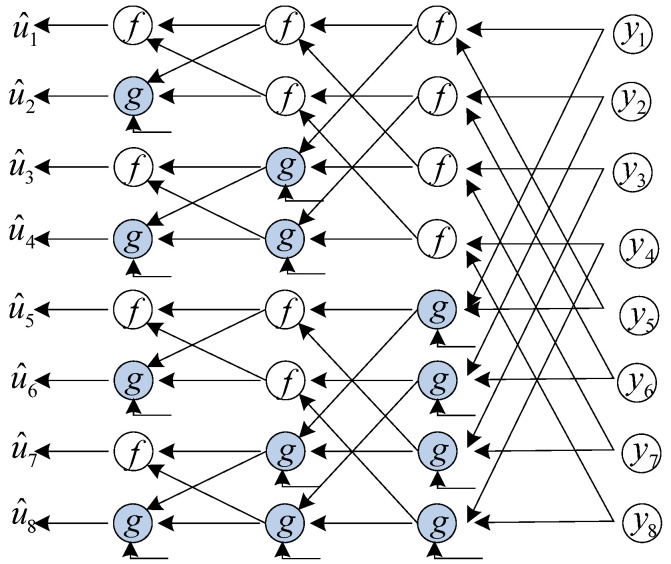
A butterfly-based decoder with N=8.

The butterfly-based decoding structure is shown in Figure 1. The SC decoding starts by computing an estimate value ${\hat{u}}_{1}$ of $u_{1}$ based only on the received value $y_{1}^{N}$. Then, $y_{1}^{N}$ and ${\hat{u}}_{i-1}$ are used to estimate ${\hat{u}}_{i}$, i=2,⋯,N. A binary input memoryless channel $W_{n}$ generates *N* sub-channels by channel splitting, defining $W_{n}^{\left(i\right)}\left(y_{1}^{N},{\hat{u}}_{1}^{i-1}|u_{i}\right)$, i=1,⋯,N. Let the LLR of $W_{n}^{\left(i\right)}\left(y_{1}^{N},{\hat{u}}_{1}^{i-1}|u_{i}\right)$ be defined as

(3) $$L_{n}^{\left(i\right)}\left(y_{1}^{N},{\hat{u}}_{1}^{i-1}|u_{i}\right)≜\log\left(\frac{W_{n}^{\left(i\right)}\left(y_{1}^{N},{\hat{u}}_{1}^{i-1}|u_{i}=0\right)}{W_{n}^{\left(i\right)}\left(y_{1}^{N},{\hat{u}}_{1}^{i-1}|u_{i}=1\right)}\right).$$

The decision of LLR can be calculated by the *f* and *g* operations in Figure 1. The *f* and *g* operations are:

(4) $$\begin{array}{c} f\left(L_{1},L_{2}\right)=\mathrm{sign}\left(L_{1}\right)\mathrm{sign}\left(L_{2}\right)\min\left(|L_{1}\left|,\right|L_{2}|\right) \end{array},$$

(5) $$\begin{array}{c} g\left(L_{1},L_{2},u\right)={\left(-1\right)}^{u}L_{1}+L_{2} \end{array},$$

where $L_{1},L_{2}\in R$ are the two inputs for the *f* and *g* operations and $u\in \left\{0,1\right\}$ is the partial sum for *g* operation. The decision of LLR is expressed as

(6) LN2i−1y1N,u^12i−2=fLNN22iy1NN22,u^1,o2i−2⊕u^1,e2i−2,LNN22iyNN2+12+1N,u^1,e2i−2,

(7) LN2iy1N,u^12i−1=gLNN22iy1NN22,u^1,o2i−2⊕u^1,e2i−2,LNN22iyNN2+12+1N,u^1,e2i−2,u^2i−1.

Finally, we obtain ${\hat{u}}_{i}$ as

(8) $${\hat{u}}_{i}=\left\{\begin{array}{cc} 0, & L_{N}^{\left(i\right)}\left(y_{1}^{N},{\hat{u}}_{1}^{i-1}\right)\geq 0\;\;\;\;and\;\;\;i\in A\\ 1, & L_{N}^{\left(i\right)}\left(y_{1}^{N},{\hat{u}}_{1}^{i-1}\right)<0\;\;\;\;and\;\;\;i\in A\;\;\\ 0, & i\in A^{C} \end{array}\right..$$

### 2.2. SC Flipping Decoder

**Figure 2:**
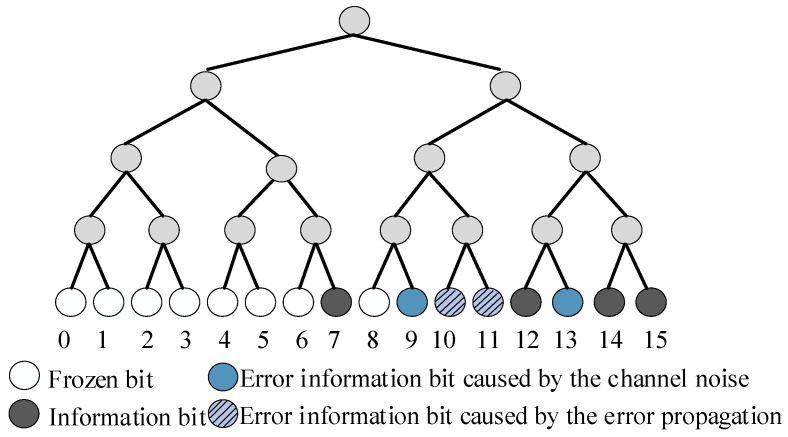
The SC decoding tree for a *P*(16, 8).

The SC flipping decoder initiates a SC decoder to generate the estimated codeword ${\hat{u}}_{1}^{N}$. If ${\hat{u}}_{1}^{N}$ passes the CRC detector, the decoding is successful. Otherwise, the flipping decoder is given *T* attempts to flip the first error bit. Algorithm 1 presents the original SC flipping algorithm, and the flipping set is denoted as V. There are two reasons for decoding failures: channel noise and error propagation. The SC flipping algorithm improves error-correction performance and restrains error propagation. However, the decoder can only eliminate the error propagation caused by the first error bit, but there may be other error bits caused by noise or follow error bits in the subsequence. The information bits {9,13} in Figure 2, for example, are error bits caused by channel noise. The SC flipping decoder tries to corrects the first error bit {9} caused by channel noise, and the error propagation phenomenon of {10,11} disappears, lowering the BER. However, {13} is still an error and the block error rate (BLER) is not lower. Therefore, we consider flipping the second error bit {13} to lower the BLER and BER in this paper.
**Algorithm 1** The SC flipping algorithm.**Input:**$y_{1}^{N}$, A, *T* **Output:**${\hat{u}}_{1}^{N}$ 1:{${\hat{u}}_{1}^{N}$, LLR} ← SC $\left(y_{1}^{N}\right)$ 2:**if**${\hat{u}}_{1}^{N}$ fail CRC and T>1 **then** 3:    V←*i*∈A of *T* smallest ∣LLR∣ 4:    **for** j←1 to *T* **do** 5:        ${\hat{u}}_{1}^{N}$ ← SC $\left(y_{1}^{N},V\left(j\right)\right)$ 6:        **if** ${\hat{u}}_{1}^{N}$ pass CRC **then** 7:           break 8:        **end if** 9:    **end for**10:**end if**11:**return**${\hat{u}}_{1}^{N}$

### 2.3. LSTM Network

**Figure 3:**
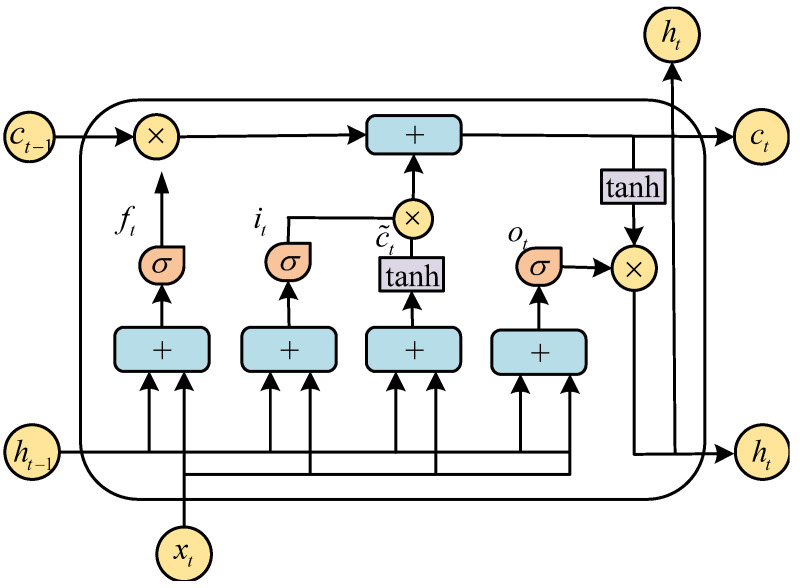
An LSTM basic unit.

Compared with the traditional Recurrent Neural Network (RNN), the LSTM network is not only capable of handling long-term dependencies but also suitable for sequence-based tasks [20]. It is well known that the SC decoding generates the LLR sequences, and the LSTM networks can effectively extract features and build relationships between sequences. The structure of LSTM is shown in Figure 3, which consists of one state and three gates, namely cell state, forget gate, input gate, and output gate. The cell state $c_{t}$ mainly saves the long-term state. The forget gate $f_{t}$ determines the proportion of the previous cell state $c_{t-1}$ retained to the current state $c_{t}$. The input gate $i_{t}$ decides the proportion of the input $x_{t}$ of the network saved to the cell state $c_{t}$. The output gate $o_{t}$ controls what message is output. The control of the three gates allows the LSTM network to remember more relevant messages for a long time, which is a great advantage when dealing with the LLR sequences. The gate is a fully connected layer, which can be expressed as:

(9) $$\begin{array}{c} \mathrm{gate}\left(x\right)=\sigma \left(\mathrm{Wx}+b\right), \end{array}$$

where W is the weight matrix, x represents the input vector, b is the bias vector, and σ is the sigmoid function:

(10) $$\begin{array}{c} \sigma \left(z\right)=\frac{1}{1+e^{-z}} \end{array}.$$

The output $h_{t}$ of the LSTM is defined by the output gate $o_{t}$ and the cell state $c_{t}$. The cell state $c_{t}$ is determined by the forgot gate $f_{t}$, the input gate $i_{t}$, the cell state $c_{t-1}$ at time t−1, and the input cell state ${c^{\prime }}_{t}$. In addition, the relationship at time *t* and t−1 is described as follows:

(11) $$\begin{array}{c} \left\{\begin{array}{c} f_{t}=\sigma \left(W_{f}\cdot \left[h_{t-1},x_{t}\right]+b_{f}\right)\\ i_{t}=\sigma \left(W_{i}\cdot \left[h_{t-1},x_{t}\right]+b_{i}\right)\\ o_{t}=\sigma \left(W_{o}\cdot \left[h_{t-1},x_{t}\right]+b_{o}\right)\\ {c^{\prime }}_{t}=\tanh\left(W_{c}\cdot \left[h_{t-1},x_{t}\right]+b_{c}\right)\\ c_{t}=f_{t}\circ c_{t-1}+i_{t}\circ c_{t}^{\prime }\\ h_{t}=o_{t}\circ \tanh\left(c_{t}\right) \end{array}\right. \end{array},$$

where tanh is the activation function defined as:

(12) $$\begin{array}{c} \tanh\left(m\right)=\frac{e^{m}-e^{-m}}{e^{m}+e^{-m}} \end{array}.$$

## 3. The Proposed SC Flipping Algorithm

**Figure 4:**
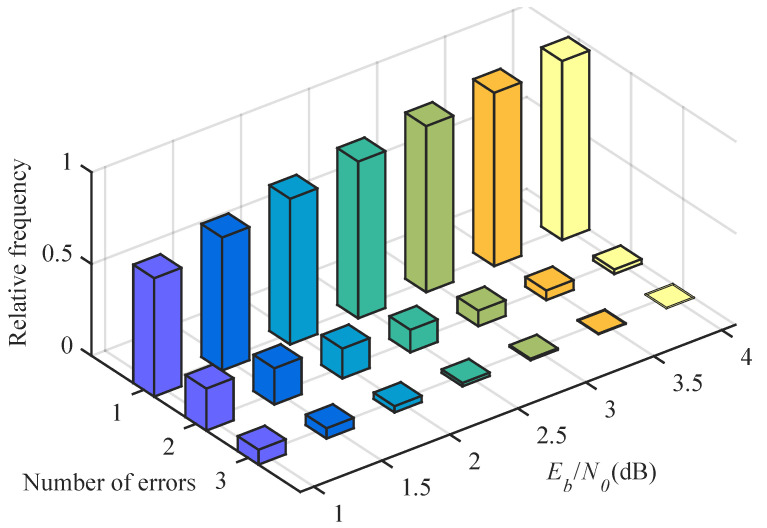
Frequency of errors caused by the channel noise at different $E_{b}$/$N_{0}$ for *P*(64, 32), $1\times 10^{5}$ polar codes.

In Figure 4, we depict the frequency of errors caused by channel noise for *P*(64, 32) at different $E_{b}$/$N_{0}$. It can be observed that the probability of one error bit occurring in the SC decoding is about 90% at high $E_{b}$/$N_{0}$, and there are still two or three error bits at low $E_{b}$/$N_{0}$. Therefore, in order to further improve the performance of the SC decoding, we also consider multi-bit flipping. In this section, we analyze the effect of error propagation. Then, we describe the DLSTM network structure and training process. Finally, we present the proposed DLSTM-based SC flipping algorithm and a mechanism to improve the robustness of the algorithm.

### 3.1. Analysis of Error Propagation

**Figure 5:**
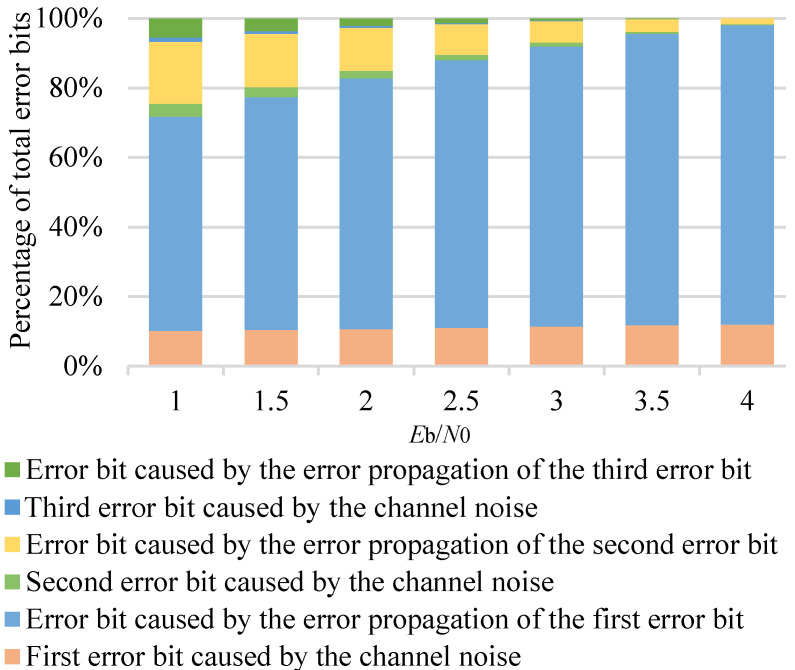
The percentage of error bits caused by different reasons at different $E_{b}$/$N_{0}$ for *P*(64, 32), $1\times 10^{5}$ polar codes.

In SC decoding, a polar code that is decided to be incorrect has multiple error bits. Figure 5 shows that the error bits caused by channel noise are much smaller than the error bits caused by error propagation. However, error propagation affects the BER, not the BLER. To improve the block error-correction performance, the error bits caused by the channel noise should be flipped. As observed in Figure 5, most of the error decisions are caused by the error propagation of the first error bit, thus it is required to construct a flipping set efficiently.

### 3.2. DLSTM Network Structure

**Figure 6:**
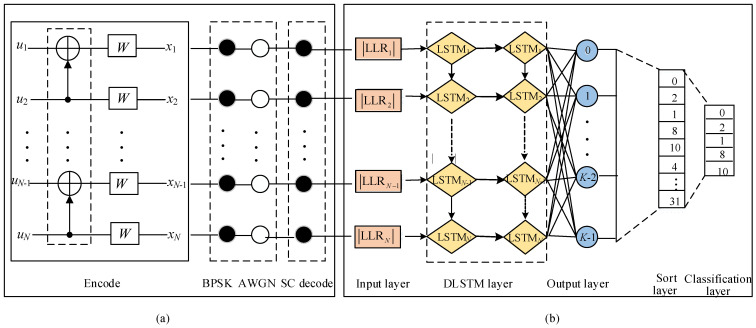
(**a**) System simulation model. (**b**) The DLSTM network structure for predicting the first error bit of P(64,32).

The first error bit plays an important role in the flipping algorithm. Since the first error bit cannot be accurately located by the LLR value, we adopt the neural network to predict it. A DLSTM network structure is illustrated in Figure 6b. This network mainly consists of an input layer, a DLSTM layer, and an output layer. The DLSTM layer contains 2N hidden LSTM units, where *N* is the length of a polar code. The output layer is a fully connected layer with *K* neurons. This *K* represents the classification that the DLSTM network can recognize, as well as the length of the information bits. The *K* neurons in output layer activated by softmax function:

(13) $$\begin{array}{c} s\left({\hat{y}}_{i}\right)=\frac{e^{{\hat{y}}_{i}}}{\sum_{j=0}^{K-1}e^{{\hat{y}}_{j}}} \end{array},$$

where ${\hat{y}}_{i}$ is the output of the *i*th neuron. With this activation function, a *K*-dimensional vector is produced.

The input and output of the DLSTM network are:

- Input: The absolute value of the LLR sequence (both the information bits and the frozen bits) that fails CRC detector in the SC decoding.
- Output: A *K*-dimensional vector, the element of which corresponds to the probability of error occurrence for each information bit.

**Figure 7:**
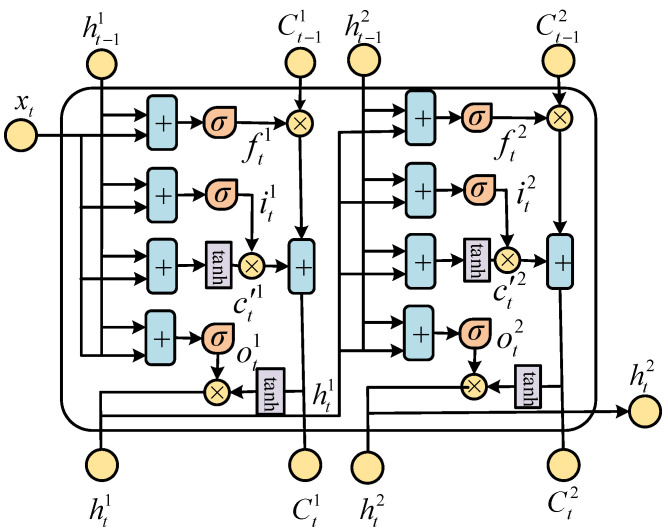
A DLSTM unit.

The details of the DLSTM unit are shown in Figure 7. At *t* time, the input of the DLSTM network is $\left[h_{t-1}^{1},x_{t}\right]$, which represents the output $h_{t-1}^{1}$ at t−1 time and the input $x_{t}$ at the current time concatenated into a vector. The vector passes through the forget gate $f_{t}^{1}$, the input gate $i_{t}^{1}$, and the output gate $o_{t}^{1}$ in turn. $h_{t}^{1}$ and $h_{t}^{2}$ denote the two outputs of the DLSTM unit. The cell states $c_{t}^{1}$ and $c_{t}^{2}$ hold the different long-term memories. The output gate $o_{t}^{1}$ of the DLSTM has two directions, one is transmitted the t+1 moment and the other is the input of the next layer at *t* moment. Thus, the input to the second LSTM layer is $\left[h_{t-1}^{2},h_{t}^{1}\right]$, which is different from the input to the first layer. The relationship between the inputs $\left[h_{t-1}^{2},h_{t}^{1}\right]$ and outputs $h_{t}^{2}$ of the second LSTM layer is described as follows:

(14) $$\begin{array}{c} \left\{\begin{array}{c} f_{t}^{2}=\sigma \left(W_{f}^{2}\cdot \left[h_{t-1}^{2},h_{t}^{1}\right]+b_{f}^{2}\right)\\ i_{t}^{2}=\sigma \left(W_{i}^{2}\cdot \left[h_{t-1}^{2},h_{t}^{1}\right]+b_{i}^{2}\right)\\ o_{t}^{2}=\sigma \left(W_{o}^{2}\cdot \left[h_{t-1}^{2},h_{t}^{1}\right]+b_{o}^{2}\right)\\ {c^{\prime }}_{t}^{2}=\tanh\left(W_{c}^{2}\cdot \left[h_{t-1}^{2},h_{t}^{1}\right]+b_{c}^{2}\right)\\ c_{t}^{2}=f_{t}^{2}\circ c_{t-1}^{2}+i_{t}^{2}\circ {c^{\prime }}_{t}^{2}\\ h_{t}^{2}=o_{t}^{2}\circ \tanh\left(c_{t}^{2}\right) \end{array}\right. \end{array}.$$

Due to this unit structure, the DLSTM network structure is more favorable than the traditional LSTM. Because of the DLSTM network’s good memory characteristic, it can show superiority in extracting historical data features and can preserve long-term relevancy.

### 3.3. Training Process of the DLSTM Network

To obtain the training data of the DLSTM network, we carried out the simulations presented in Figure 6a. First, we obtained the polar codes according to the method in Section 2. Then, we performed the binary phase shift keying (BPSK) demodulation and incorporated additive white Gaussian noise (AWGN) channel noise. After that, $2\times 10^{6}$ polar codes were generated and decoded by the SC decoder. If the decoding result passed the CRC detector, the decoding was successful. Otherwise, it became the training sample for the DLSTM network. The sample consists of the absolute value of the LLR sequence and one-hot encoding of the first error bit, which is denoted as $D=\{\left(x_{1},y_{1}\right),\left(x_{2},y_{2}\right),\cdots ,\left(x_{j},y_{j}\right)\}$, where *j* is the size of the sample, including the training set and validation set. $x_{c}$, 1≤c≤j is the input of the network. $y_{c}$, 1≤c≤j is the label and represents the expected output of the DLSTM network. The process of extracting information bits is shown in Table 1, where the binary sequence in the second row is the information pattern, in which ‘0’ denotes the frozen bit and ‘1’ denotes the information bit. The proposed algorithm clips all frozen bits in the output layer because flipping a frozen bit is useless and causes corresponding error propagation. The classification is reduced by (N−K)/N compared to the method of Author17 [17].

In order to train a more efficient DLSTM network, the data were divided into the training set and validation set according to the ratio of 8:2. During the training, the regularization method [21] was used to prevent training overfitting. The L2-norm was applied to the weights, taking L2 = 0.008. The Adam optimizer [22] was adopted to design adaptive learning rates for different parameters because it can handle sparse gradients very well. The goal of the DLSTM network is to be a well-trained classifier where each information bit is a category. We employed the cross-entropy function to measure the loss between the expect value and the predicted value.

(15) $$\begin{array}{c} E\left(x\right)=-y\log\hat{y}+\left(1-y\right)\log\left(1-\hat{y}\right) \end{array},$$

where *y* represents the labeled data and $\hat{y}$ represents the predicted value of the network. The smaller is the loss *E*, the better is the network trained. As the entire DLSTM is trained offline, it does not increase the SC flipping decoder complexity.

### 3.4. DLSTM-Based SC Flipping Algorithm

**Figure 8:**
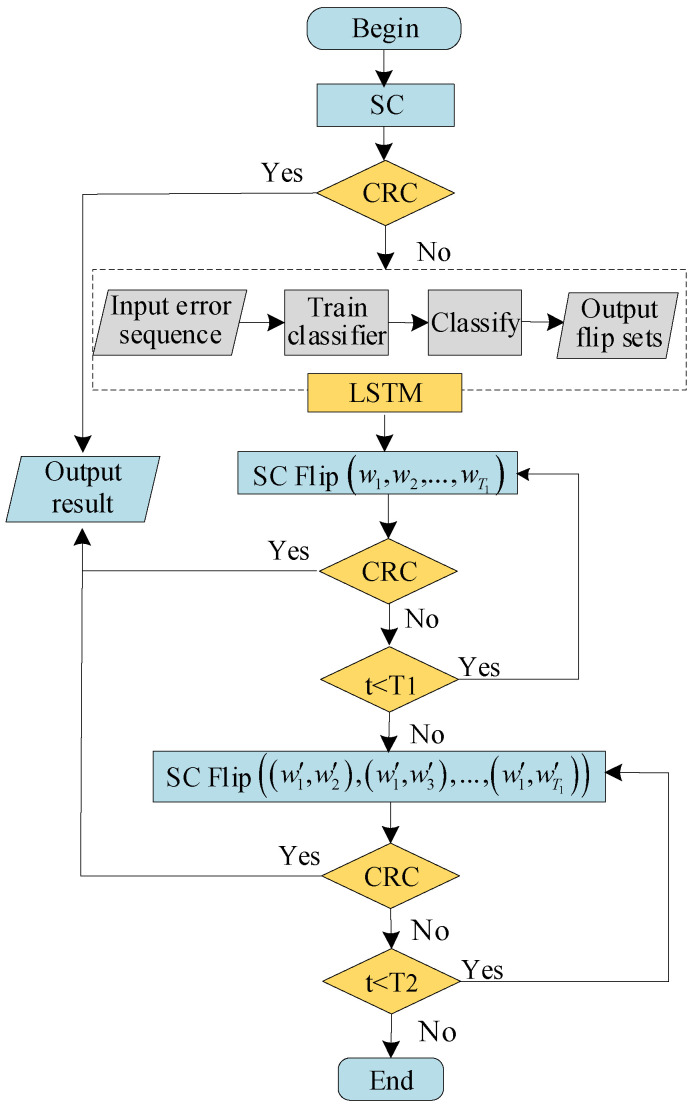
The SC Flipping algorithm based on DLSTM.

We utilized the well-trained DLSTM network to determine the flipping set of the first error bit. According to Author23 [23], the channels reliability ordering can be used to determine the flipping positions in SC flipping decoder. Therefore, we adopted channel reliability to determine the multiple error bits. This method avoids the ordering of the LLRs, which reduces the complexity of SC flipping decoder. Figure 8 illustrates the process of the DLSTM-based SC flipping algorithm, which consists of the following steps.

**Figure 9:**
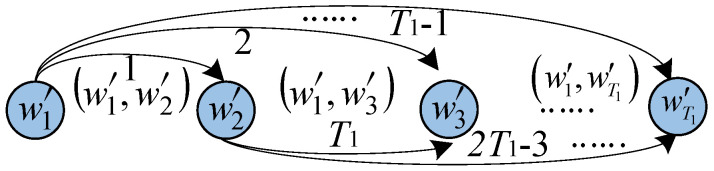
Diagram of two-bit flipping selection.

(1) Perform the SC decoding and check whether the decoding result can pass the CRC detector. If pass, the decoding is successful. Otherwise, go to Step 2.

(2) Take advantage of the well-trained DLSTM to predict the probability of error occurring in the information bits. Then, sort their probabilities in descending order. Select the index of the $T_{1}$ maximum probability and map them to the corresponding locations of the information bits. $T_{1}$ is the maximum number of one-bit flipping and is also the size of the flipping set. Employ these locations to construct the flipping set $W_{1}^{T}=\left(w_{1},w_{2},\cdots ,w_{T_{1}}\right)$, where $w_{1},w_{2},\cdots ,w_{T_{1}}$ are the candidate bits for the first error bit and the probability $w_{1}>w_{2}>\cdots >w_{T_{1}}$, and go to Step 3.

(3) Select $w_{1}$ for flipping. If the decoding fails, flip the following bits $w_{2},w_{3},\cdots ,w_{T_{1}}$ successively. If $T_{1}$ attempts are unsuccessful, then go to Step 4. Otherwise, terminate the decoding.

(4) Calculate the channel reliability of flipping set $W_{1}^{T}$ by Gaussian approximation. For $W_{1}^{T}=\left(w_{1},w_{2},\cdots ,w_{T_{1}}\right)$, reorder into $W_{2}^{T}=\left(w_{1}^{\prime },w_{2}^{\prime },\cdots ,w_{T_{1}}^{\prime }\right)$ in ascending order according to reliability, where the reliability follows: $w_{1}^{\prime }<w_{2}^{\prime }<\cdots <w_{T_{1}}^{\prime }$. Select $w_{1}^{\prime }$ and $w_{2}^{\prime }$ from $W_{2}^{T}$ to perform bit flipping at the same time. If the CRC detector fails, next select $\left(w_{1}^{\prime },w_{3}^{\prime }\right)$, $\left(w_{1}^{\prime },w_{4}^{\prime }\right)$, …, $\left(w_{T_{1}-1}^{\prime },w_{T_{1}}^{\prime }\right)$ in turn to flip. If $T_{2}$ attempts are unsuccessful, the decoding fails, where $T_{2}$ is the maximum number of two-bit flipping. Otherwise, the decoding is successful. The selection method is shown in Figure 9.

The pseudocode for two-bit flipping algorithm based on the DLSTM network is shown in Algorithm 2. It can be extended to multi-bit flipping in a similar way. The maximum number of candidate bits is *K*. The set of multi-bit flipping is denoted as $W_{3}^{T}$, $W_{4}^{T}$, …, $W_{K}^{T}$. When simultaneously flipping the least reliable 3, 4, …, *K* bits, the maximum number of flips is indicated as $T_{3}$, $T_{4}$, …, $T_{K}$.
**Algorithm 2** Two-bit flipping algorithm based on the DLSTM.**Input:**$y_{1}^{N}$, $W_{1}^{T}$, $W_{2}^{T}$, $T_{1}$, $T_{2}$ **Output:**${\hat{u}}_{1}^{N}$ 1:{${\hat{u}}_{1}^{N}$,LLR} ← SC $\left(y_{1}^{N}\right)$ 2:$t_{1}=0$, $t_{2}=0$ 3:**while**${\hat{u}}_{1}^{N}$ fail CRC and $t_{1}<T_{1}$ **do** 4:    $W_{1}^{T}$ ← DLSTM 5:    ${\hat{u}}_{1}^{N}$ ← SC flip $\left(y_{1}^{N},W_{1}^{T}\left(t_{1}\right)\right)$ 6:    **if** ${\hat{u}}_{1}^{N}$ pass CRC **then** 7:        break 8:    **else** 9:        $t_{1}\leftarrow t_{1}+1$10:    **end if**11:**end while**12:**if**$t_{1}\geq T_{1}$**then**13:    $t_{1}=0$14:    **while** ${\hat{u}}_{1}^{N}$ fail CRC **do**15:        **for** $i\leftarrow t_{1}+1$ to $T_{1}$ **do**16:           **if** $t_{2}<T_{2}$ **then**17:               ${\hat{u}}_{1}^{N}$ ← SC flip $\left(y_{1}^{N},W_{2}^{T}\left(t_{1},i\right)\right)$18:               **if** ${\hat{u}}_{1}^{N}$ pass CRC **then**19:                   break20:               **else**21:                   $t_{2}\leftarrow t_{2}+1$22:               **end if**23:           **end if**24:        **end for**25:        $t_{1}\leftarrow t_{1}+1$26:    **end while**27:**end if**28:**return**${\hat{u}}_{1}^{N}$

### 3.5. DLSTM-Based Robustness Mechanism

**Figure 10:**
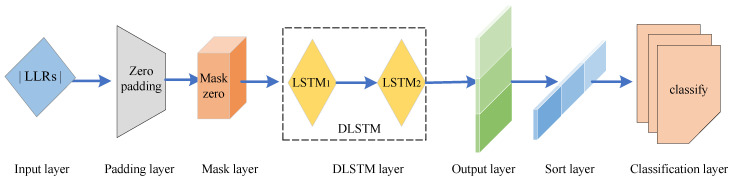
Structure of the DLSTM-based robustness algorithm.

Current the neural network decoders are specific, such as certain block length or signal-to-noise ratios (SNR). To make the algorithm robust, we designed a DLSTM network architecture that is compatible with multiple block lengths. For different block length polar codes $P_{1}\left(N_{1},K_{1}\right)$, $P_{2}\left(N_{2},K_{2}\right)$, $P_{3}\left(N_{3},K_{3}\right)$, and $N_{1}>N_{2}>N_{3}$, we make the following updates to maintain the integrity of the input shape. The length $N_{1}$ of the longest codewords is taken as the standard, and padding is performed for codewords smaller than this length. One-hot encoding is done directly for labels with length $K_{1}$. After making the length uniform for polar codes with variable length, the padding part is learned during the training, which affects the prediction results. To reduce the impact, a mask layer is added before the DLSTM layer for skipping the respective time step. During the DLSTM training, the part that is masked is not involved in the calculation. Figure 10 presents this training process. To obtain better generalization capability, we adopt an early stopping criterion. The training is stopped if the accuracy of the validation set does not improve.

## 4. Performance Analysis

In this section, we first give the DLSTM network training results. Then, we evaluate the decoding delay, complexity, and error-correction performance of the proposed algorithm and other existing algorithms. Simulations were performed with AWGN channel and BPSK modulation on an Intel(R) Xeon(R) Gold 6132 CPU @ 2.60 GHz server.

### 4.1. DLSTM Network Training Results

The DLSTM network was trained by the LLR sequences and the one-hot encoding of first error bit. The simulation parameters for P(64,32) are shown in Table 2. As described in Section 2, a basic LSTM unit consists of three gates and one cell state. Each gate and cell state is a fully connection layer with *N* (hidden size) neurons. Thus, a layer of LSTM actually has four fully connection layers. Equation (9) can also be written as

(16) $$\begin{array}{c} \mathrm{gate}\left(x_{t}\right)=\sigma \left(W\cdot \left[h_{t-1},x_{t}\right]+b\right) \end{array}.$$

Therefore, one layer of the basic LSTM network has 4($h_{s}$($h_{s}+x_{d}$)+$h_{s}$) parameters, where $h_{s}$ is the size of the hidden layer and $x_{d}$ is the dimension of the input data. As shown in Table 2, a layer of basic LSTM has $4\times \left[64\times \left(64+64\right)+64\right]$ = 33,024 parameters. Thus, a layer of DLSTM has 2× 33,024 = 66,048 parameters. Dense denotes the fully connection layer. The *N*(hidden size)-dimensional vector output from the DLSTM network is used as the input to the dense layer, so the dense layer has (h_s·K+K) parameters, where *K* is the number of neurons in the dense layer.

**Figure 11:**
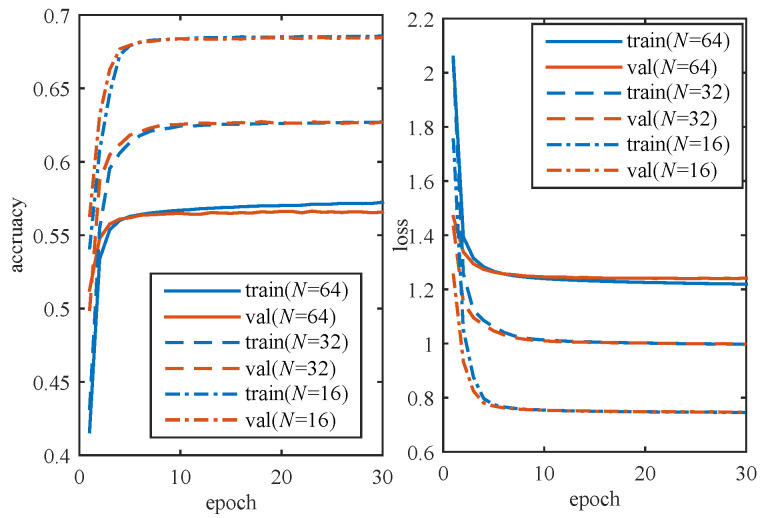
Convergence graph for different codewords with $E_{b}/N_{0}$ = 1.0 dB.

All the data are trained once for one epoch. In each epoch, the network calculates the accuracy and loss of the training set and validation set. After 30 epochs, the accuracy and loss changes for different codewords with $E_{b}/N_{0}$ = 1.0 dB are depicted in Figure 11. It can be seen that, before five epochs, the accuracy of the validation set is higher than that of the training set. As the number of epoch increases, the accuracy of the training set starts to increase and outperform that of the validation set. Finally, the accuracy of both the validation set and training set converge to a stable value. It is observed that the DLSTM network converges and reaches a stable state after about 20 epochs.

**Figure 12:**
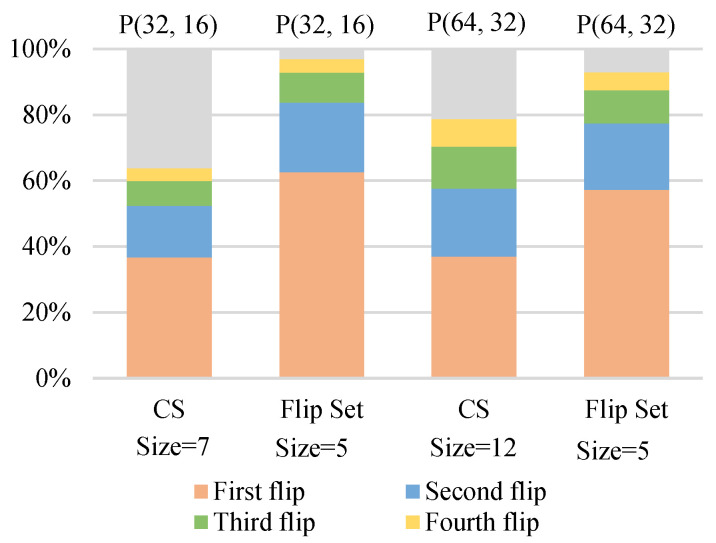
A comparison of the CS in [8] and the flipping set in this paper for identifying the first error bit at $E_{b}/N_{0}$ = 1.0 dB.

**Figure 13:**
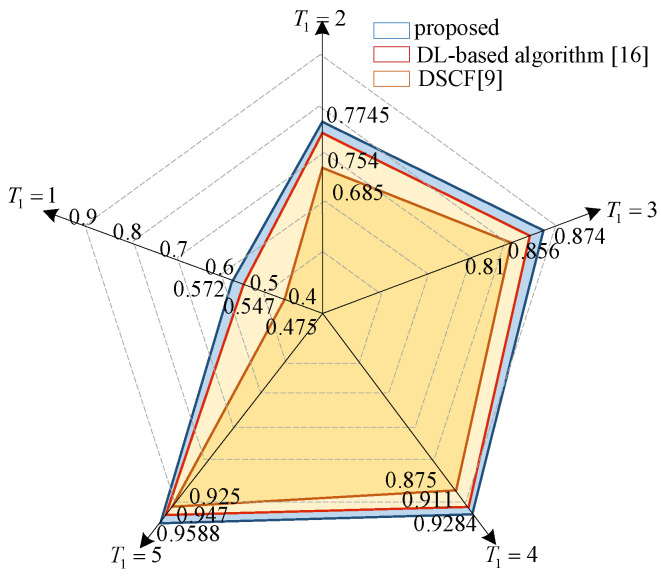
Accuracy comparison of identifying the first error bit for P(64,32) at $E_{b}/N_{0}$ = 1.0 dB.

In this paper, a flipping set of size $T_{1}$ is constructed for one-bit flipping. Figure 12 shows that, when the number of flips is smaller than the size of the CS, our flipping set has a higher accuracy in identifying the first error bit compared to the CS in [8]. Although CS contains the first incorrect hard decision with high probability, the simulation results show that superior error correction performance is achieved when the number of flips is comparable to the size of CS. Figure 13 also illustrates the comparison of identifying the first error bit at $E_{b}/N_{0}$ = 1.0 dB. The proposed algorithm has higher accuracy than the other two algorithms within the same number of flips. At the first flip, the accuracy of the proposed algorithm is improved by 2.5% and 9.7% compared to the DL-based algorithm and the DSCF decoder, respectively. Table 3 displays the comparison of the two- and three-layer LSTMs. The accuracy of the two-layer network in predicting the first error bit is comparable, but the training parameters of the DLSTM network are reduced by about 1/3. Therefore, it is appropriate to train with a DLSTM network, which can effectively reduce the training complexity.

### 4.2. Decoding Complexity and Latency Analysis

**Figure 14:**
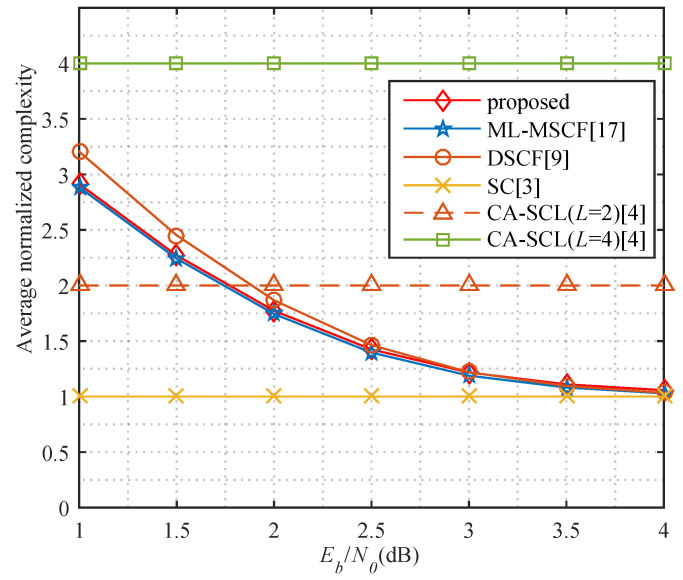
Comparison of average normalized decoding complexity for *P*(64, 32) with $T_{1}=4,T_{2}=2$.

The SC flipping decoder starts with the SC decoder. The complexity of SC is O(NlogN). In constructing the flipping set, the complexity of the sorting operation on *K* probabilities is O(KlogK). When flipping one bit, there are no more than $T_{1}$ additional attempts, and the complexity is $O(T_{1}N\log N)$. When flipping two bits, the $T_{1}$ candidate bits are sorted according to channel reliability with a complexity of $O(T_{1}\log T_{1})$. Then, no more than $T_{2}$ additional attempts are performed, and the complexity is $O(T_{2}N\log N)$. Finally, if $T_{1}>T_{2}$, the maximum complexity of the proposed algorithm is $O(T_{1}N\log N)$. Otherwise, the maximum complexity is $O(T_{2}N\log N)$. However, the flipping is only for the error sequence that fails the CRC detector, so the average complexity is related to the BLER. Therefore, the average complexity of the proposed algorithm is $O(\left(1+P_{e}\left(T_{1}+T_{2}\right)\right)N\log N+K\log K+T_{1}\log T_{1})$, where $P_{e}$ is the BLER of the SC decoding. As shown in Figure 14, the average decoding complexity is high because $P_{e}$ is dramatically large at low $E_{b}/N_{0}$. However, $P_{e}$ decreases rapidly as $E_{b}/N_{0}$ increases, and the average complexity rapidly decreases, finally converging to the complexity of the SC decoding. It can be noticed that the average decoding complexity is lower than the SCL decoding (*L* = 4) and the DSCF decoding and slightly higher than the ML-MSCF decoding.

**Figure 15:**
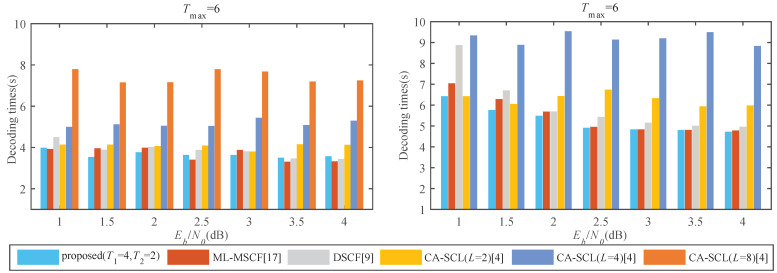
(**left**) Comparison of total decoding latency for *P*(32, 16). (**right**) Comparison of total decoding latency for *P*(64, 32).

It takes a lot of time to train an effective DLSTM network, but the whole training is done offline and can benefit from some acceleration platforms [24]. Therefore, the training process does not increase the decoding latency. However, the prediction time is calculated as part of the total decoding latency. Compared with the traditional approach, the prediction is an additional computational process. In Figure 15, the decoding latency represents the total time consumed for decoding as calculated by the CPU. The proposed algorithm consumes comparable time to the ML-MSCF decoder. When $E_{b}/N_{0}$ = 1.0 dB, the consumed time is decreased by 11.41% and 27.68% compared to the DSCF decoder for both code lengths, which saves a lot of computation time by avoiding exponential and logarithmic operations, respectively. Compared to CA-SCL (*L* = 2), the consumed time is lowered by 3.64% at *N* = 32 but comparable at *N* = 64. Compared with CA-SCL (*L* = 4), the time consumption is reduced by 20.32% and 31.26%, respectively, and the time to try *L* decoding paths is reduced.

### 4.3. BER and BLER Analysis

**Figure 16:**
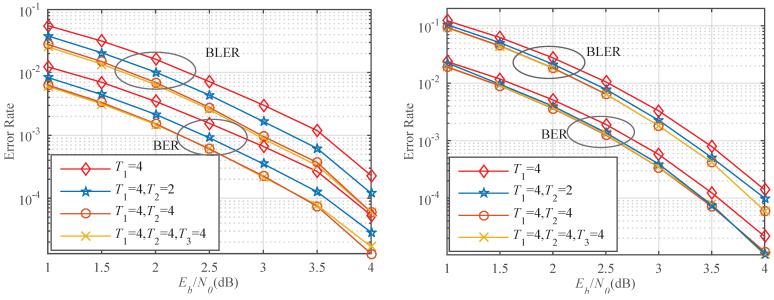
(**left**) Performance comparison of different flip times for *P*(32, 16). (**right**) Performance comparison of different flip times for *P*(64, 32).

In Figure 16 left, we compare the error-correction performance for the proposed algorithm with $T_{1}=4$, $T_{2}=2$, 4, and $T_{3}=4$ for *P*(32, 16). The proposed decoder adopts a 4-bit CRC and its generator polynomial is $g\left(x\right)=x^{4}+x+1$. It can be noticed that, with continuous flipping, a lot of gain can be achieved. When BLER = $10^{-3}$, the proposed algorithm with $T_{1}=4$, $T_{2}=2$, and $T_{1}=4$, $T_{2}=4$ can achieve gains of approximately 0.32 dB and 0.57 dB than that with $T_{1}=4$, respectively. At BER = $10^{-4}$, the proposed algorithm with $T_{1}=4$, $T_{2}=2$, and $T_{1}=4$, $T_{2}=4$ can obtain performance improvement of about 0.25 and 0.46 dB compared to that with $T_{1}=4$, respectively. The error-correction performance of the proposed algorithm with $T_{1}=4$ and $T_{2}=4$ is close to that with $T_{1}=4$, $T_{2}=4$, and $T_{3}=4$. This is due to the occurrence of three errors with low frequency. In Figure 16 right, the block length is changed to 64. The proposed algorithm with $T_{1}=4$ and $T_{2}=4$ is lower than that with $T_{1}=4$, $T_{2}=2$, and $T_{1}=4$ in terms of the BLER and the BER. The proposed algorithm for *N* = 32 has a significant performance gain compared to that for *N* = 64. On the one hand, the shorter is the block length, the higher is the prediction accuracy. On the other hand, the degree of channel polarization for *N* = 64 is better than for *N* = 32. A polar code with *N* = 64 has fewer error bits than that with *N* = 32.

**Figure 17:**
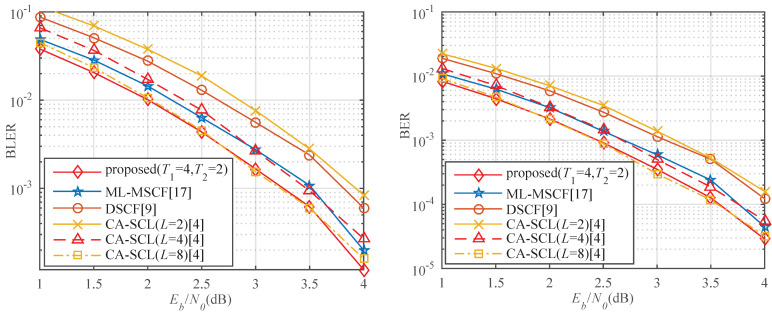
(**left**) BLER comparison of different algorithms for *P*(32, 16). (**right**) BER comparison of different algorithms for *P*(32, 16).

Figure 17 left shows the BLER performance of the proposed algorithm compared with the existing algorithms for *P*(32, 16) with $T_{\max}=6$. It can be seen that the proposed algorithm significantly outperforms the ML-MSCF decoder and the DSCF decoder within the same number of flips. It is also more efficient than CA-SCL (*L* = 2, *L* = 4). It is close to the performance of CA-SCL (*L* = 8). As shown in Table 4, the proposed algorithm can achieve approximately 0.28 and 0.49 dB performance gain, but the decoding latency is increased by 5.77% and 1.16% over the ML-MSCF decoder and the DSCF decoder at BLER = $10^{-3}$. Compared with CA-SCL (*L* = 2, *L* = 4), it can obtain performance gain of about 0.66 and 0.19 dB, and the decoding latency is reduced by 15.73% and 31.22%, respectively. The performance is slightly lower than that of CA-SCL (*L* = 8), but the decoding latency is decreased by 51.46%. In Figure 17 right, compared with the ML-MSCF decoder, DSCF decoder, and CA-SCL (*L* = 2, *L* = 4), the proposed algorithm has lower BER.

**Figure 18:**
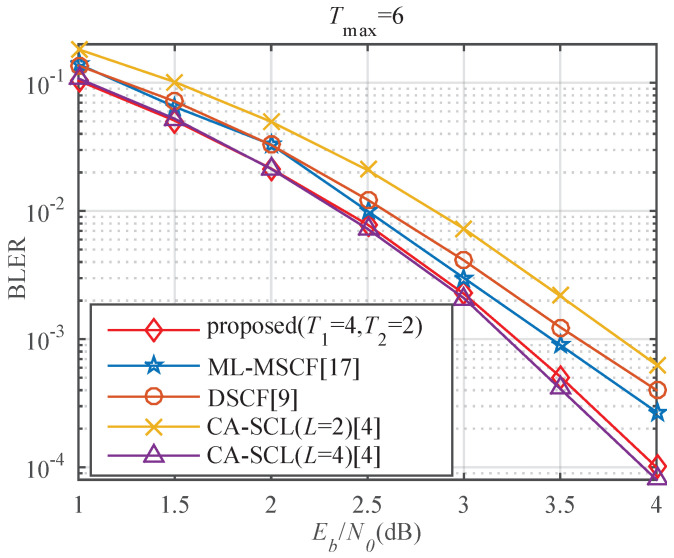
BLER comparison of different algorithms for *P*(64, 32).

Figure 18 compares the BLER performance for *P*(64, 32) with $T_{\max}=6$. It can be noticed that the proposed algorithm has better error-correction performance than the ML-MSCF decoder, DSCF decoder, and CA-SCL (*L* = 2). It is close to the performance of CA-SCL (*L* = 4). Table 5 shows the obtained gains and reduced latency using the proposed algorithm over other algorithms at BLER = $10^{-3}$. The proposed algorithm achieves a gain of about 0.21 dB compared to the ML-MSCF decoder, but the decoding latency is slightly increased. Compared with the DSCF decoder and the CA-SCL (*L* = 2) decoder, the proposed algorithm can obtain gains of about 0.36 and 0.55 dB, and decoding latency is reduced by 1.39% and 19.29%, respectively. The performance is slightly lower than that of CA-SCL (*L* = 4), but the decoding latency is decreased by 45.41%. This is due to multiple additional attempts when flipping one bit and selecting the most least reliable position for multi-bit flipping based on channel reliability. When the code length is 32, the proposed algorithm can approach the performance of CA-SCL (*L* = 8), but, when the code length is 64, it is close to the performance of CA-SCL (*L* = 4). This is because the accuracy of the proposed algorithm for N=32 is higher than that for N=64.

### 4.4. BLER Analysis of the Algorithm with Robustness Mechanism

**Figure 19:**
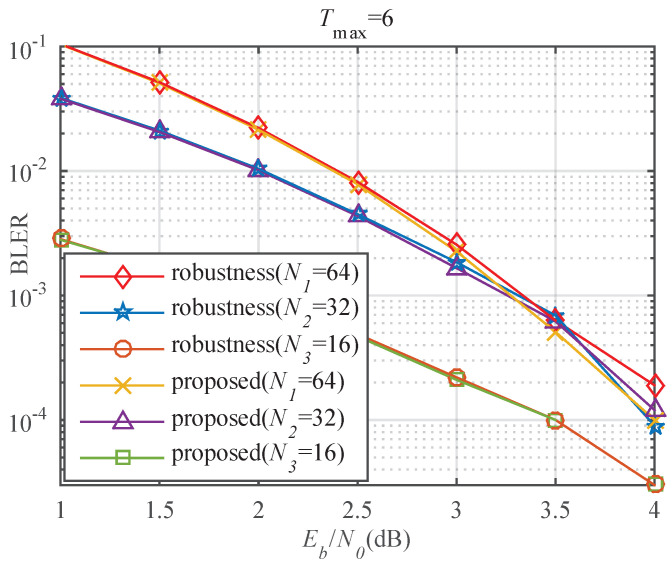
BLER comparison of the proposed algorithm and optimized algorithm with robustness mechanism with $T_{\max}=6$.

The proposed algorithm with robustness mechanism is compatible with different block lengths ($N_{1}=64$, $N_{2}=32$, and $N_{3}=16$). The proposed decoder for $N_{3}=16$ with a 2-bit CRC has a generator polynomial of $g\left(x\right)=x^{2}+1$. Figure 19 demonstrates that the BLER performance of the proposed algorithm and optimized algorithm is close for the three block lengths. The optimized algorithm has generalization ability and can break the block length constraint during neural network training. The large-scale processing capability of the neural network is also exploited.

## 5. Conclusions

To improve the performance of short block length, this paper proposes a DLSTM-based SC flipping algorithm for short polar codes. The DLSTM is used to predict the first error bit and construct a flipping set for the one-bit flipping, clipping all frozen bits to enhance the accuracy of prediction. For the sequences that fail CRC detector, the flipping set is resorted according to channel reliability, and the most unreliable bits are selected for multi-bit flipping. The simulation results show that the proposed algorithm has better error-correction performance compared with the ML-MSCF decoder and the DSCF decoder. In addition, when $E_{b}/N_{0}$ = 1.0 dB, the decoding performance is close to CA-SCL (*L* = 8) for *N* = 32, and the decoding latency is reduced by 49.03%. Furthermore, to make the algorithm robust, we propose a mechanism to make it compatible with different block lengths. For future work, we will focus on multi-task neural networks to predict multiple error bits of polar codes simultaneously.

## Figures and Tables

**Table 1:** Extract information bits for *P*(16, 8).

Index	0	1	2	3	4	5	6	7	8	9	10	11	12	13	14	15
Location	0	0	0	0	0	0	0	1	0	1	1	1	1	1	1	1
Index of information bit								0		1	2	3	4	5	6	7

**Table 2:** Simulation parameter for *P*(64, 32) with $E_{b}/N_{0}$ = 1.0 dB.

Name	Parameter
Polar codes	(64,32)
Frame number	2,000,000
Rate R	1/2
CRC generator polynomial	$x^{8}+x^{7}+x^{5}+x^{4}+x^{1}+1$
Batch size	1000
Number of epoch	30
Regularization L2	0.008
DLSTM	66,048
Dense	2080
Optimizer	Adam

**Table 3:** Comparison of two- and three-layer LSTM networks in predicting the first error bit.

Polar Codes	Layer	Accuracy				Total Param
P(64,32)	2	57.2%	77.45%	87.4%	92.84%	68,128
	3	57.12%	77.32%	87.43%	92.94%	101,152
P(16,8)	2	68.63%	91.8%	98.48%	99.86%	4360
	3	68.46%	91.81%	98.44%	99.85%	6472

**Table 4:** The obtained gain and reduced latency by the proposed algorithm for *P*(32, 16) with $T_{\max}=6$ and BLER = $10^{-3}$.

Algorithm	$E_{b}/N_{0}$	Gain (dB)	Reduce Latency
proposed	3.25	-	-
ML-MSCF [17]	3.53	0.28	−5.77%
DSCF [9]	3.74	0.49	−1.16%
CA-SCL (*L* = 2) [4]	3.91	0.66	15.73%
CA-SCL (*L* = 4) [4]	3.44	0.19	31.22%
CA-SCL (*L* = 8) [4]	3.23	−0.02	51.46%

**Table 5:** The obtained gain and reduced latency by the proposed algorithm for *P*(64, 32) with $T_{\max}=6$ and BLER = $10^{-3}$.

Algorithm	$E_{b}/N_{0}$	Gain (dB)	Reduce Latency
proposed	3.26	-	-
ML-MSCF [17]	3.47	0.21	−0.62%
DSCF [9]	3.62	0.36	1.39%
CA-SCL (*L* = 2) [4]	3.81	0.55	19.29%
CA-SCL (*L* = 4) [4]	3.24	−0.02	45.41%

## Data Availability

Not applicable.

## Abbreviations

The following abbreviations are used in this manuscript:

5G	Fifth generation
SC	Successive cancellation
DLSTM	Double long short term memory
CRC	Cyclic redundancy check
LLR	Log-likelihood ratio
CS	Critical set
BER	Bit error rate
BLER	Block error rate
BPSK	Binary phase shift keying
AWGN	Additive white Gaussian noise
